# Supplementary material for: Achieving Order in Disorder: Stabilizing Red Light-Emitting α-Phase Formamidinium Lead Iodide
Source: Nanomaterials (Basel). 2023 Nov 29;13(23):3049. doi: 10.3390/nano13233049 (PMC10708465; doi:10.3390/nano13233049)
Supplement: Supplementary file 1 [file nanomaterials-13-03049-s001.zip › nanomaterials-2736066-supplementary.pdf]

Supplementary Materials for

Achieving Order in Disorder: Stabilizing Red Light-Emitting α-Phase Formamidinium Lead Iodide

Aditya Narayan Singh [1], Atanu Jana [2], Manickam Selvaraj [3], Mohammed A. Assiri [3], Sua Yun [4] and Kyung-Wan Nam [1,4,5,*]

[1] Department of Energy and Materials Engineering, Dongguk University—Seoul, Seoul 04620, Republic of Korea; aditya@dongguk.edu
[2] Division of Physics and Semiconductor Science, Dongguk University, Seoul 04620, Republic of Korea; atanujanaic@gmail.com
[3] Department of Chemistry, Faculty of Science, King Khalid University, Abha 61413, Saudi Arabia; mselvaraj@kku.edu.sa (M.S.); maassiri@kku.edu.sa (M.A.A.)
[4] Department of Advanced Battery Convergence Engineering, Dongguk University—Seoul, Seoul 04620, Republic of Korea; suanet50@gmail.com
[5] Center for Next Generation Energy and Electronic Materials, Dongguk University—Seoul, Seoul 04620, Republic of Korea
* Correspondence: knam@dongguk.edu

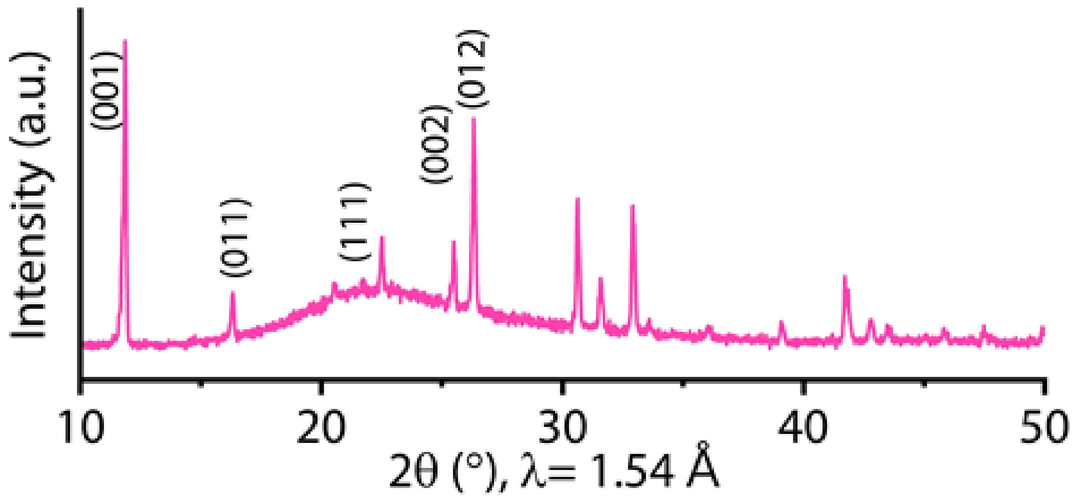

Figure S1. Crystal structure determination of δ-$FAPbI_3$.

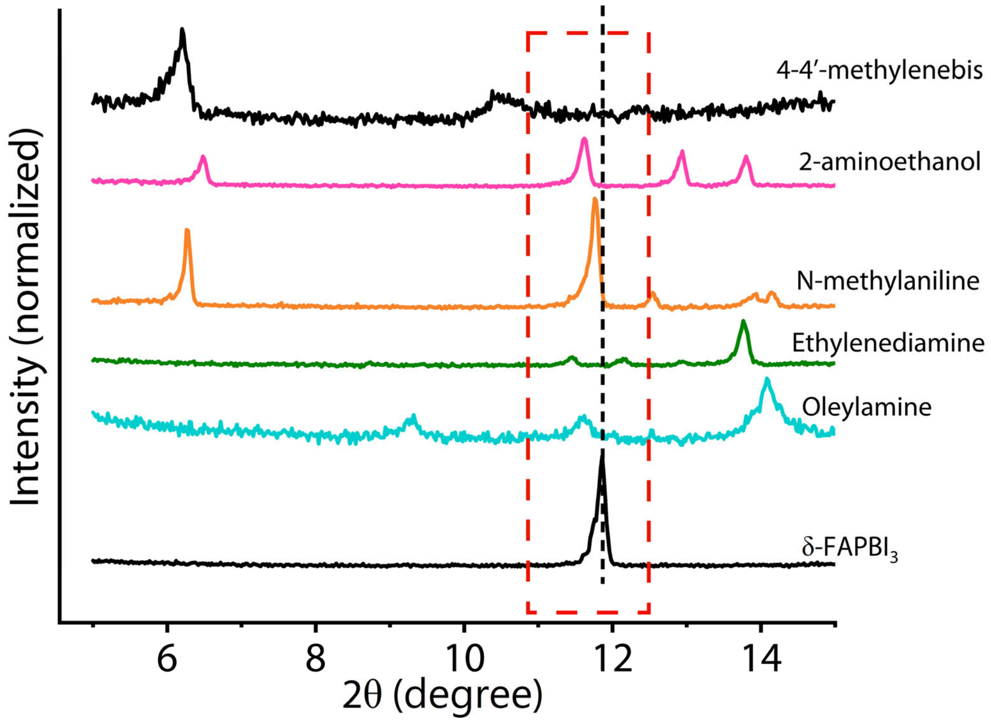

Figure S2. Zoomed version of XRD showing lower angle shifting.

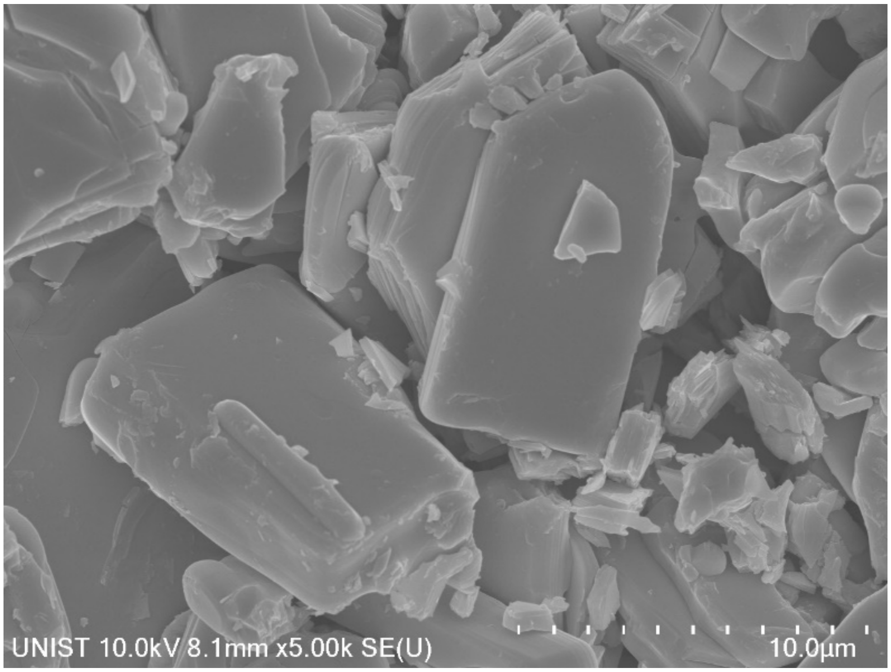

Figure S3. Lower magnification image under SEM.

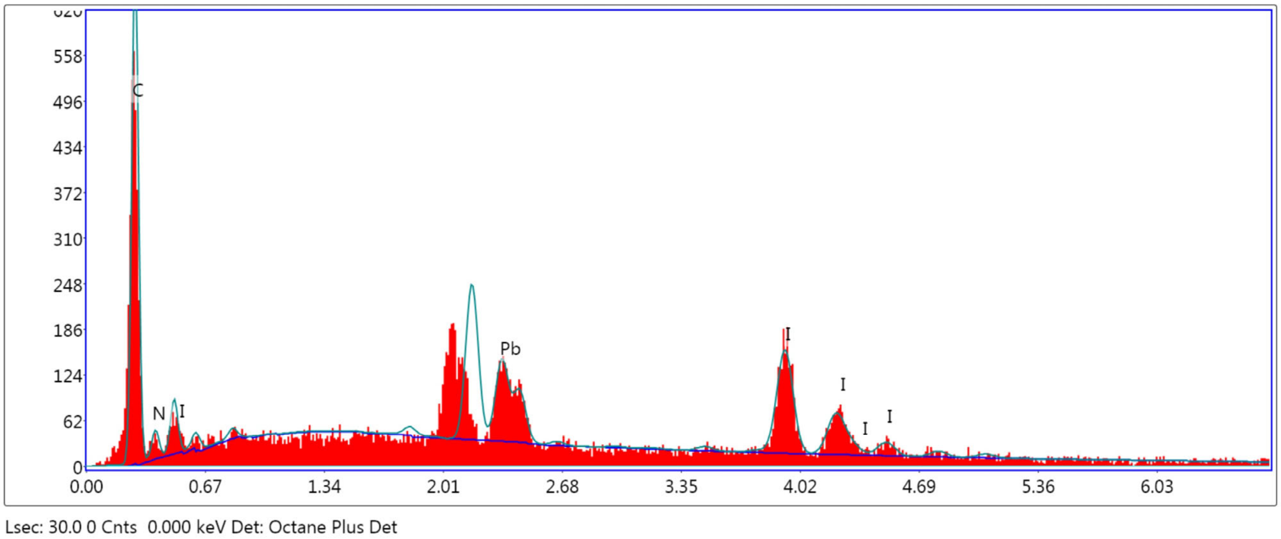

Figure S4. EDX mapping of ethylenediamine.

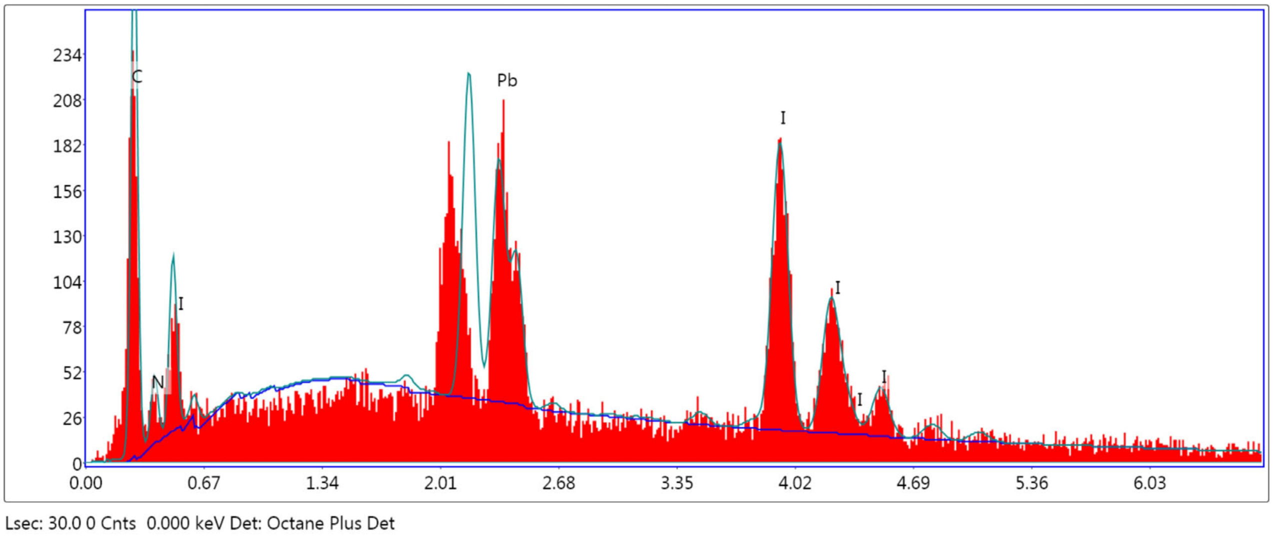

Figure S5. EDX mapping of N-methylaniline.

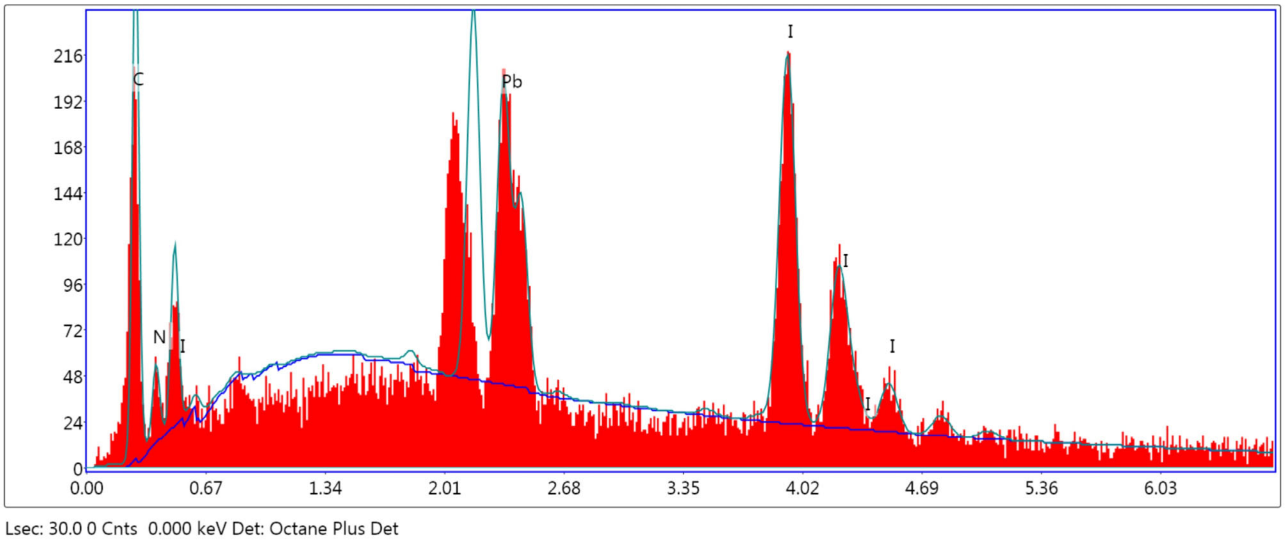

Figure S6. EDX mapping of 2-aminoethanol.

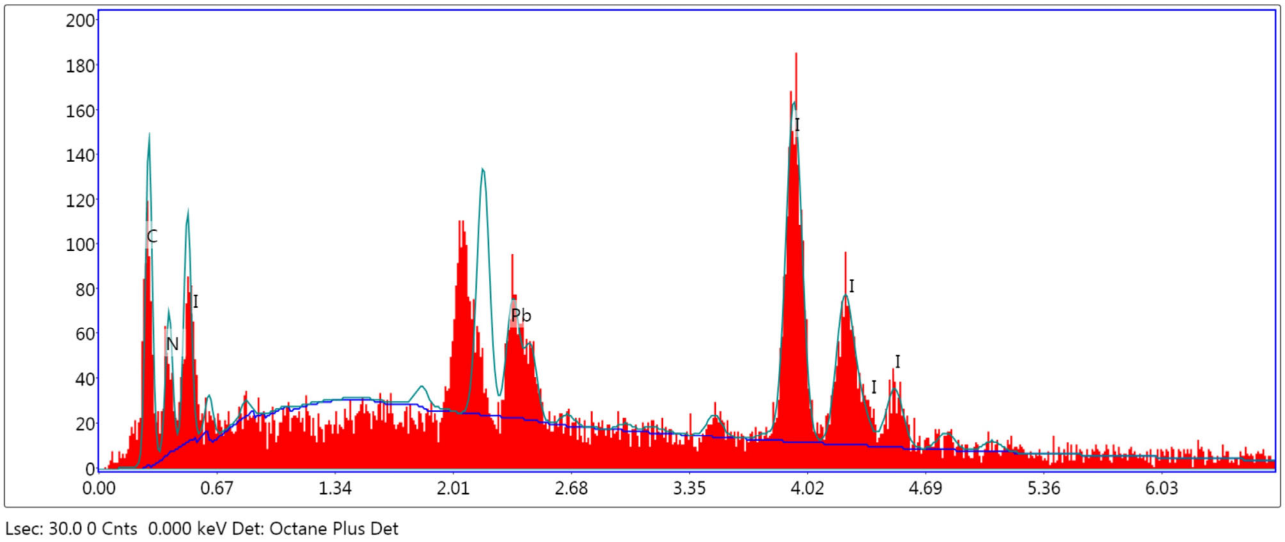

Figure S7. EDX mapping of 4-4′-methylenebis.

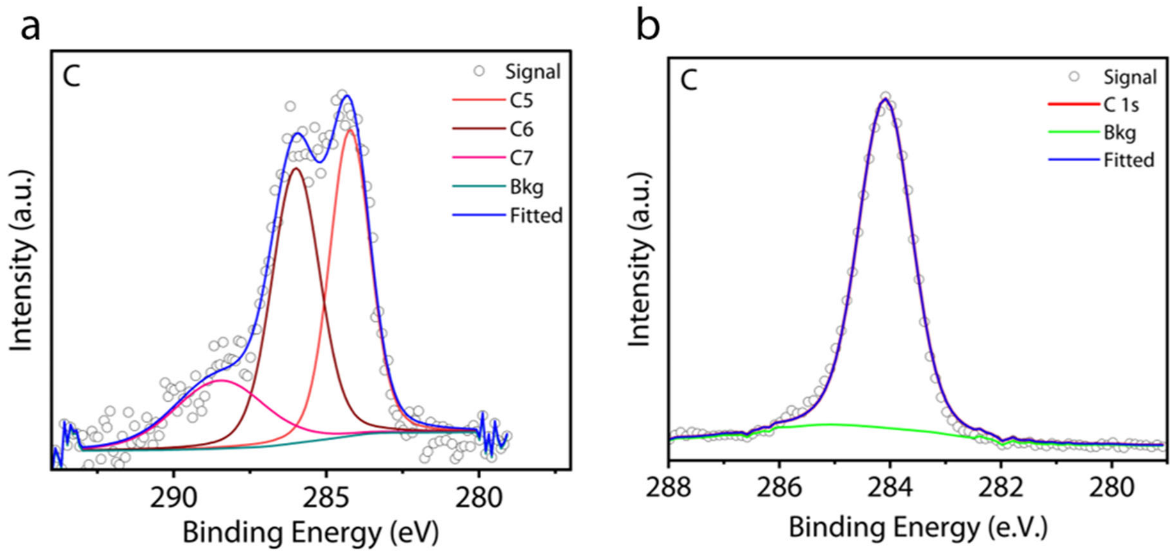

Figure S8. XPS spectra for (a) δ-FAPbI3. (b) α-FAPbI₃ using oleylamine.

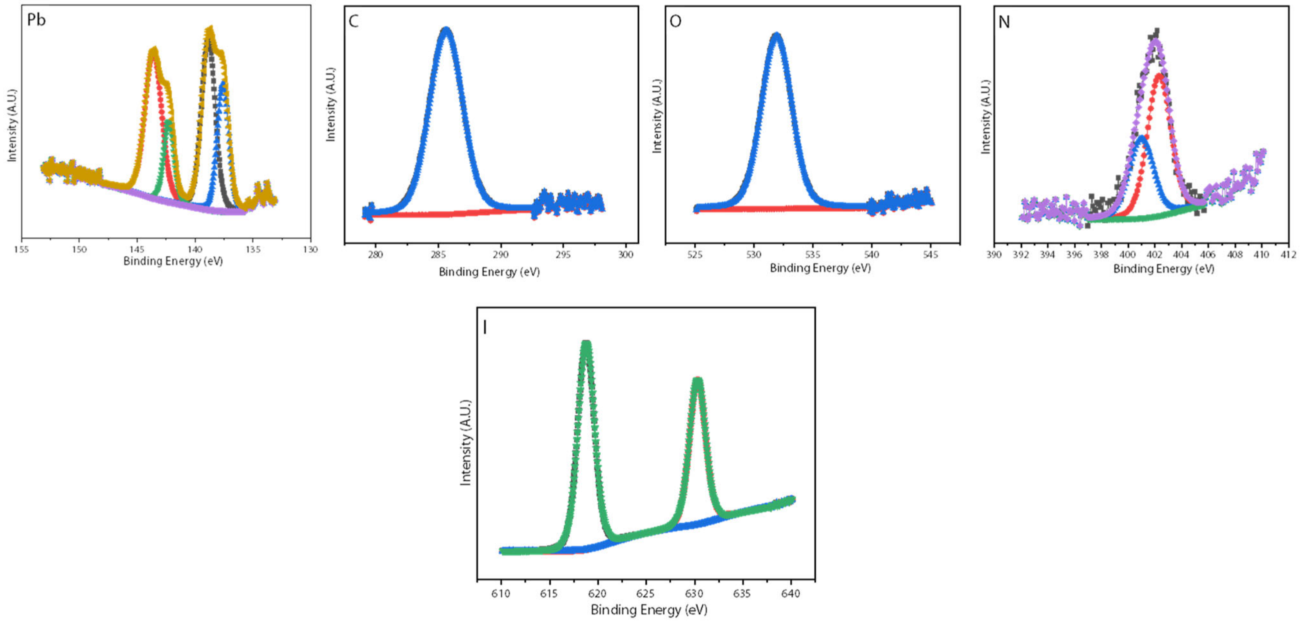

Figure S9. XPS spectra for ethylenediamine.

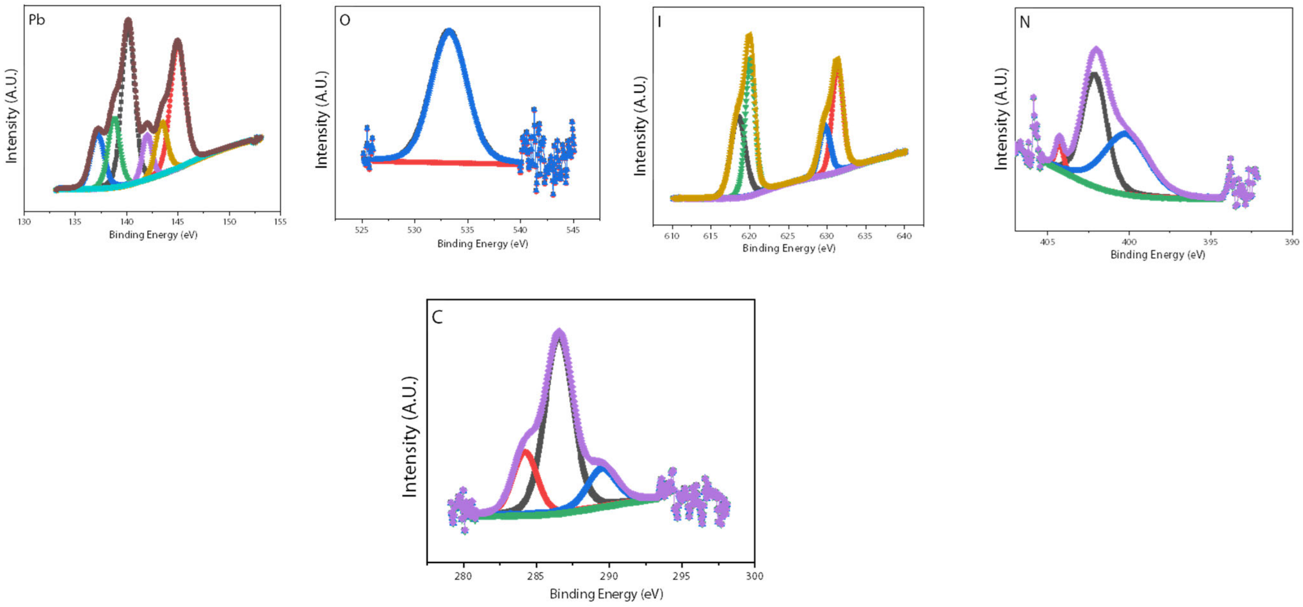

Figure S10. XPS spectra for N-methylaniline.

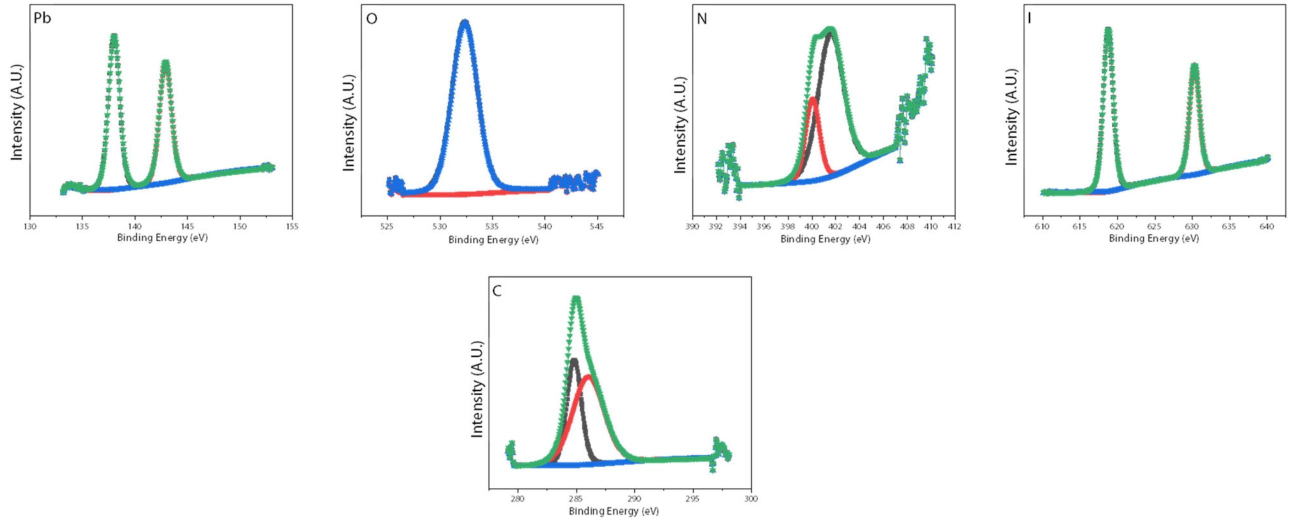

Figure S11. XPS spectra for 2-aminoethanol.

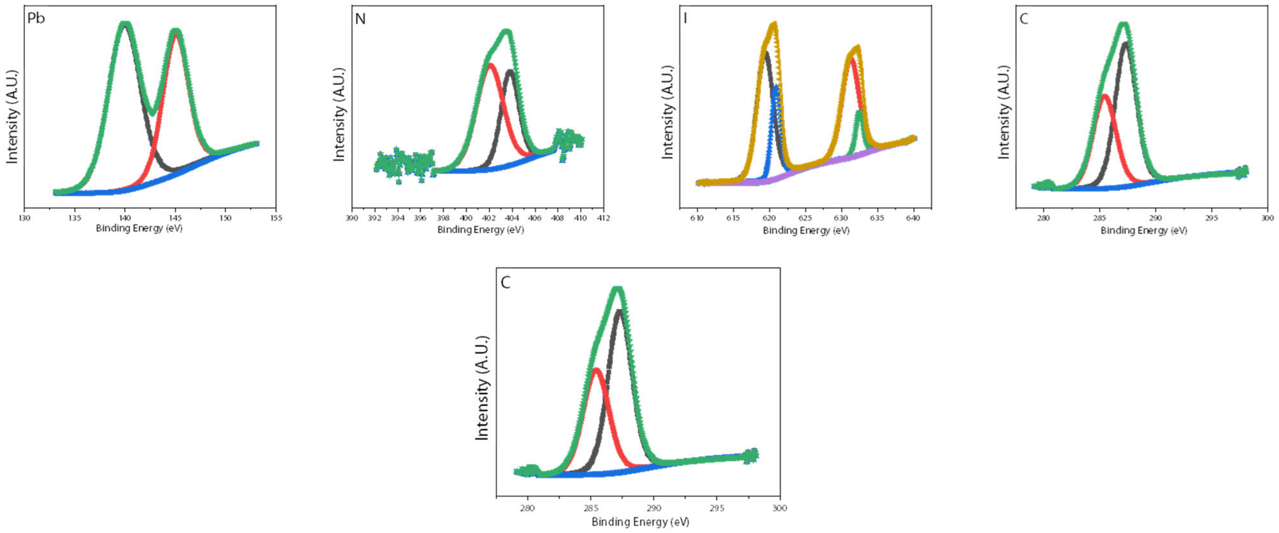

Figure S12. XPS spectra for 4-4′-methylenebis.

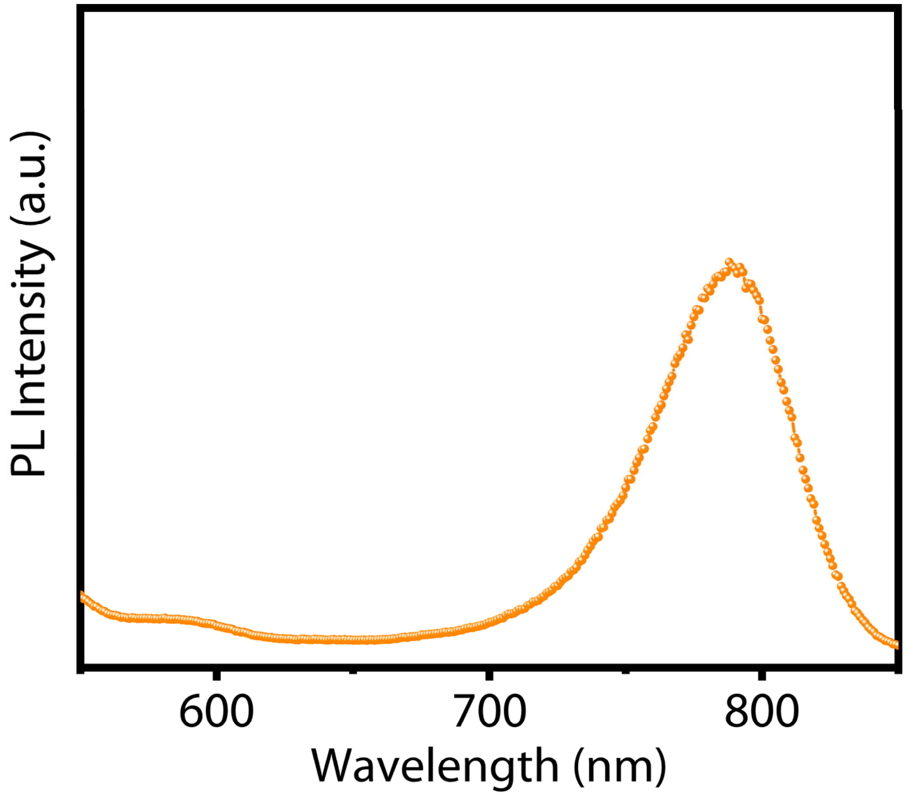

Figure S13. Emission spectra for 4-4′-methylenebis.

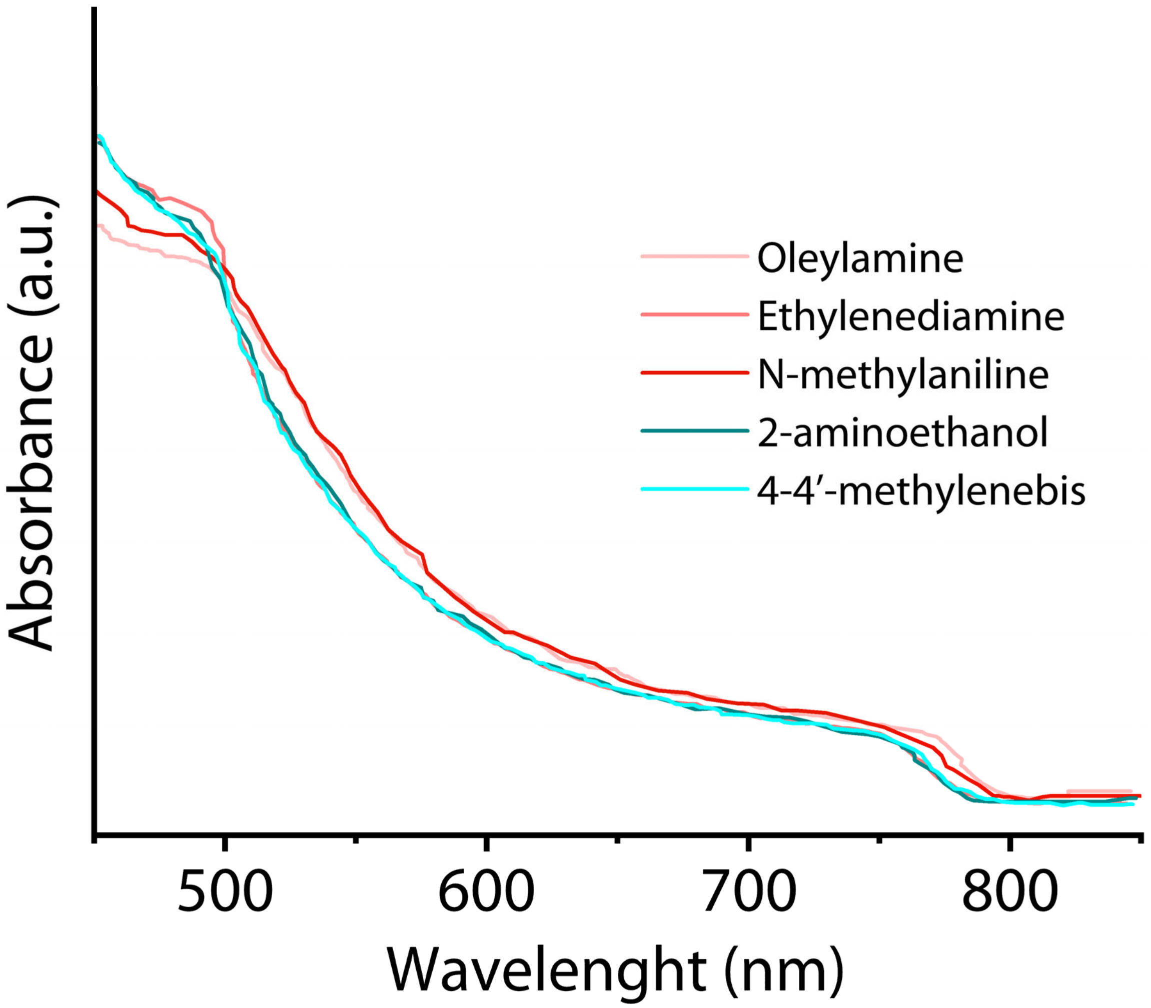

Figure S14.UV-Vis spectra.
